# Nox4 Facilitates TGFβ1-Induced Fibrotic Response in Human Tenon’s Fibroblasts and Promotes Wound Collagen Accumulation in Murine Model of Glaucoma Filtration Surgery

**DOI:** 10.3390/antiox9111126

**Published:** 2020-11-13

**Authors:** Manisha H. Shah, Elsa C. Chan, Nicole J. Van Bergen, Surinder S. Pandav, Sze Ng, Jonathan G. Crowston, Hitesh M. Peshavariya

**Affiliations:** 1 Centre for Eye Research Australia, Royal Victorian Eye and Ear Hospital, Victoria 3002, Australia; elsa.chan@unimelb.edu.au (E.C.C.); nicole.vanbergen@mcri.edu.au (N.J.V.B.); Sspandav@yahoo.com (S.S.P.); calavera1984@y7mail.com (S.N.); crowston@unimelb.edu.au (J.G.C.); hitesh.peshavariya@unimelb.edu.au (H.M.P.); 2 Department of Medicine, University of Melbourne, Melbourne 3010, Victoria, Australia; 3 Centre for Vision Research, Duke-NUS, Singapore 169856, Singapore; 4 Singapore Eye Research Institute/ Singapore National Eye Centre (SERI/ SNEC), Singapore 169856, Singapore; 5 Department of Ophthalmology, University of Melbourne, Melbourne 3010, Victoria, Australia

**Keywords:** collagen, glaucoma filtration surgery, NADPH Oxidase, transforming growth factor-beta, reactive oxygen species

## Abstract

Collagen accumulation in sub-conjunctival tissue at the surgical wound is one of the major complications associated with glaucoma filtration surgery (GFS). This process often leads to unwanted fibrotic scar formation at the lesion site and dysfunction of tissues. Previously, we demonstrated that NADPH oxidase 4 (Nox4) is implicated in transforming growth factor-beta (TGFβ)-induced collagen production in ocular fibroblasts and scarring responses in a mouse model of corneal injury. Here, we propose that Nox4 is an important facilitator of TGFβ-induced responses. We tested this hypothesis in human Tenon’s fibroblasts (HTF) and also assessed a role of Nox4 in an experimental mouse model of GFS. TGFβ1 induced Nox4 mRNA expression but downregulated Nox5 in HTF. Targeting Nox4 gene expression with an adenovirus carrying a Nox4 small interfering RNA (siRNA) (Ad-Nox4i) or removal of hydrogen peroxide (H_2_O_2_) with EUK-134 (25 μM) in HTFs significantly reduced TGFβ1-induced Nox4 expression, H_2_O_2_ production, and collagen synthesis (*p* < 0.05, *n* = 3–6). SIS3 (5 μM) that prevents Smad3 phosphorylation is found to suppress TGFβ1-induced collagen production in HTFs. Furthermore, Ad-Nox4i and EUK-134 both abolished TGFβ1-stimulated proliferation of HTFs. We also compared collagen deposition at the wound arising from GFS between wildtype (WT) and Nox4 knockout (KO) mice. Both collagen deposition and fibrovascularization at the wound were significantly decreased in Nox4 KO mice at 14 days after GFS. Our results provide comprehensive evidence that Nox4 is an important mediator for TGFβ1-induced responses in HTFs and collagen deposition in surgical wound following GFS in mice. As such, pharmacological inhibition of Nox4 would be a viable therapeutic strategy for the control of scarring after glaucoma surgery.

## 1. Introduction

Glaucoma is the leading cause of vision impairment and blindness [1], and it results from degeneration of the optic nerve, often exacerbated by increasing pressure within the eye (intraocular pressure (IOP)). If IOP lowering drugs or laser treatment fails, glaucoma filtration surgery (GFS) is the gold standard operation widely used to treat glaucoma and manage IOP by creating an opening that bypasses the trabecular meshwork and allows aqueous to drain into the sub-conjunctival space [2]. However, trabeculectomy procedure can sometimes stimulate excessive scarring and fibrosis in the sub-conjunctival tissue, which is the principal cause of GFS failure and inadequate control of IOP [3]. To reduce the risk of bleb scarring, anti-mitotic agents mitomycin C (MMC) and fluorouracil (5-FU) are often applied intra-operatively or post-operatively [4,5] to inhibit fibroblasts activation and improve surgical outcomes. However, the cytotoxic nature of these drugs poses limitation and risk on their applications as an adjunct to GFS. A safer and more effective means has always been explored to improve the long-term success of GFS.

GFS is known to activate a key cell type identified as Tenon’s fibroblasts in the conjunctiva. Activation of these cells enhances the proliferation and production of fibrotic proteins, leading to excess scar formation in the bleb [6]. One of the major factors involved in bleb scarring is transforming growth factor (TGF) β, and increased levels of TGFβ1 and β2 have indeed been detected in the filtering bleb from patients after GFS [7,8]. Anti-TGFβ2 strategy with monoclonal antibody has failed to prevent ocular scarring in patients with glaucoma surgery [9]. Moreover, TGFβ is a key profibrotic factor, it has crucial physiological functions and hence, direct inhibition of TGFβ may have severe off-target effects. As such, intervention at signaling proteins/molecules downstream of TGFβ may be a better therapeutic approach for preventing post-surgical scarring.

Recently, we and others have shown that TGFβ-dependent fibrotic responses in cultured fibroblasts or animal models of fibrosis involved NADPH oxidase 4 (Nox4) [10,11,12,13]. Nox4 is an isoform of the NADPH oxidase enzyme that is known to solely produce reactive oxygen species (ROS), such as superoxide and hydrogen peroxide (H_2_O_2_) [14]. An oral treatment with a Nox4 inhibitor substantially reduced the extent of fibrosis and expression of TGFβ-activated fibrotic markers, such as collagen and fibronectin, in a preclinical model of bleomycin-induced lung fibrosis [15], hence highlighting a role of Nox4 in TGFβ and lung fibrosis. Although the mechanisms underlining TGFβ-dependent fibrotic responses are common in lung and ocular fibrosis, it remains unclear if Nox4 contributes to collagen deposition in wound healing following GFS surgery. Therefore, we hypothesized that TGFβ induces scarring responses via a Nox4-mediated signaling pathway, and inhibition of Nox4 activity will suppress fibrotic responses in human Tenon’s fibroblasts (HTFs). We have, therefore, tested this hypothesis in vitro using primary cultures of human Tenon’s fibroblasts (HTF). Using Nox4 knockout (KO) mice, we have further assessed the role of Nox4 in post-surgical scarring in an experimental model of GFS.

## 2. Materials and Methods

### 2.1. Human Tenon’s Fibroblast Culture

As previously described, HTFs were derived from explanted subconjunctival Tenon’s capsule collected during GFS performed in patients [16]. The patients included for collecting the human biopsy specimens were aged between 18–65 years who were booked for the first glaucoma filtration procedure with no history of a clinically significant inflammatory eye condition and no recent anterior surgery, such as cataract extraction and lens implantation. Explanted tissue derived from patients (*n* = 3) were attached to the bottom of a six-well plate with a sterile coverslip and overlaid with Roswell Park Memorial Institute Medium (RPMI)-1640 supplemented with 10% fetal bovine serum (FBS), penicillin (100 U/mL) and streptomycin (100 μg/mL) (all from Invitrogen, Victoria, Australia). Once the monolayers reached confluence, fibroblasts were then propagated, passaged, and subcultured in 75-cm^2^ tissue culture flasks. Cells were incubated at 37 °C/5% CO_2_ in a humidified incubator. Independent experiments were performed with different passages of cells. The collection of human biopsy specimens was conducted in accordance with The Code of Ethics of the World Medical Association (Declaration of Helsinki). Institutional human ethics committee (St. Vincent’s Hospital Human Research Ethics Committee (HREC 07/724H) approval was granted and written informed consent was obtained from all participating patients.

### 2.2. Pharmacological Drug Treatment

Unless otherwise specified, HTFs were serum starved for 24 h, followed by TGFβ1 induction (5 ng/mL) for 6 h for H_2_O_2_ measurement, gene expression, and Western blot analysis, and 24 h for total collagen assay. In some cases, the cells were pre-treated with Smad3 inhibitor SIS3 (5 μM; Sigma-Aldrich, New South Wales, Australia) or superoxide dismutase (SOD)/catalase mimetic EUK-134 (25 μM; Cayman Chemical, Ann Arbor, MI, USA) for 30 min prior to adding TGFβ1 (5 ng/mL; Sigma-Aldrich, New South Wales, Australia).

### 2.3. Adenovirus Infection

As described in our previous studies, expression of Nox4 gene was silenced using adenoviral vectors expressing small interfering RNA (siRNA). In brief, siRNA that has been designed to target human Nox4 nucleotides 418–436 from the start codon (Ad-Nox4i) [17] was used to infect cells to suppress the ROS production capacity of Nox4. Adenovirus expressing green fluorescent protein siRNA (Ad-CtrlRNAi-GFP) was used as a control. HTFs (10^5^ cells/well) were seeded in 6-well plate at one day prior to infection. The HTFs were then infected with 2500 multiplicity of infection (MOI) of either Ad-CtrlRNAi or Adv-Nox4i in reduced serum Opti-MEM medium (Life Technologies, Waltham, MA, USA) for 24 h. HTFs were then allowed to recover in complete RPMI medium for another 24 h. All experiments were then carried out at 48 h after infection.

### 2.4. Determination of Extracellular H_2_O_2_

Extracellular level of H_2_O_2_ from cells were analyzed using Amplex^®^ Red assay kit (Molecular Probes, Life Technologies, Victoria, Australia) according to manufacturer’s instructions as described previously [10]. Briefly, cells (2 × 10^4^ cells/well) were seeded in a 24-well plate and incubated overnight at 37 °C/5% CO_2_. Cells were treated with or without TGFβ1 (5 ng/mL) and in combination with EUK-134 (10 µM) or adenoviruses for 24 h in Krebs-Ringer Bicarbonate Buffer containing 0.1% serum, Amplex^®^ Red reagent (50 µM), and horseradish peroxidase (HRP; 0.1 U/mL). Fluorescence was then measured for 30 min at 37 °C using a Polarstar microplate reader (BMG Labtech, Ortenberg, Germany) with excitation and emission wavelengths at 550 nm and 590 nm, respectively. 

### 2.5. Gene Expression Analysis

HTFs (10^5^ cells/well) were plated in 6-well plates and incubated overnight at 37 °C/5% CO_2_. Cells were serum deprived for 24 h, followed by treatment with inhibitors and/or TGFβ1. Total RNA was extracted from treated cells using the TRI Reagent according to manufacturer’s protocol (Ambion, Waltham, MA, USA). cDNA was prepared from 200 ng of total RNA using high capacity performance reverse transcription reagents (Applied Biosystems, Waltham, MA, USA) at 25 °C for 10 min, at 37 °C for 2 h, and followed by 85 °C for 5 s in a Thermal cycler (BioRad-DNA Engine, Bio-Rad, Hercules, CA, USA). The quantitative real-time PCR (RT-PCR) reactions were performed (7300 system, Applied Biosystems, Life Technologies, Carlsbad, CA, USA) using TaqMan Universal PCR master mix and commercially available predesigned gene-specific probes and primer sets (TaqMan Gene Expression Assay, Life Technologies, Victoria, Australia) for Nox1, Nox2, Nox4, and Nox5 and GAPDH (Glyceraldehyde 3-phosphate dehydrogenase ) sequence (Table 1). Diethyl pyrocarbonate (DEPC)-treated water was used as a negative control. The cycle threshold (CT) values from all quantitative rt-PCR experiments were analyzed using ^ΔΔ^CT method. Data were normalized to housekeeping gene GAPDH and expressed as fold changes over that in the control group.

### 2.6. Western Blot Analysis

HTFs (1.5 × 10^5^ cells/well) were cultured in 6-well tissue culture plates, serum deprived for 24 h, and followed by TGFβ1 induction. Proteins was extracted in radioimmunoprecipitation assay buffer RIPA lysis buffer and equal amounts of protein were then separated by electrophoresis using gradient SDS-PAGE gels and transferred to nitrocellulose membranes (Amersham Pharmacia, GE Healthcare Biosciences Pty. Ltd., New South Wales, Australia). After blocking non-specific proteins with 5% non-fat skim milk in Tris–HCl (20 mM, pH 7.5), NaCl (100 mM) and Tween 20 (0.1%) buffer respective membranes were incubated at 4 °C overnight with either primary rabbit monoclonal anti-NOX4 (No. ab60940, lot# GR18955-1; 1:1000 dilution, Abcam, Victoria, Australia), rabbit monoclonal anti-phospho-Smad2/Smad3 (No. 8828, 1:1000; Cell Signaling Technology, Danvers, MA, USA), or rabbit monoclonal anti-total Smad2/3 (No. 8685, 1:1000; Cell Signaling Technology, Danvers, MA, USA) and normalized with housekeeping mouse monoclonal anti-β-actin (No. ab8224, 1:10,000, Merck Millipore, Darmstadt, Germany) antibodies. Proteins were detected using an enhanced chemiluminescence detection kit (GE Healthcare, New South Wales, Australia) with horseradish peroxidase conjugated to appropriate secondary antibodies (Bio-Rad, New South Wales, Australia). The image was captured and processed using CanoScan 8800F/PhotoStudio 5.5 software (New South Wales, Australia).

### 2.7. Picro-Sirius Red Spectrophotometric Assay

Total collagen content was measured using Sirius red based high-throughput assay. Briefly, HTF cells (2.5 × 10^4^) seeded in 96-well plates followed by various treatments of inhibitors with or without TGFβ1 were fixed using methanol for 1 h at −20 °C, followed by a gentle wash with phosphate-buffered saline PBS to prevent cell loss, and then incubated in Picro-Sirius red (0.1%; Sigma-Aldrich, Australia) for 1 h at room temperature. Picro-Sirius red was then removed, and cells were washed three times with 0.1% acetic acid. Picro-Sirius red was then eluted in 0.1 N sodium hydroxide (NaOH), 200 µL/well, the plates were placed on a rocking platform at room temperature for 1 h, and the optical density at 540 nm was determined using a Bio-Tek spectrophotometer (BioTek, Winooski, VT, USA).

### 2.8. Cell Proliferation Assay

Cells (10^4^ cells/well) were plated in a 24-well plate. Serum-deprived cells were treated with or without EUK-134 (10 µM) for 24 h. Cell proliferation was then induced by replacing serum-free media with RPMI containing 0.5% serum. After 48 h, cell numbers were analyzed using Alamar blue assay kit. Each well was incubated with Alamar blue assay solution (1:10 dilution with RPMI media; Life Technologies, Victoria, Australia) for 1 h at 37 °C, 5% CO_2_. Fluorescence was then measured with excitation and emission wavelengths of 480 nm and 520 nm, respectively, using a Polarstar microplate reader at 37 °C.

### 2.9. Mouse Model of Glaucoma Filtration Surgery

All procedures were performed as per National Institutes of Health guide for the care and use of Laboratory animals and were approved by the institutional animal care and use committee (St. Vincent’s Animal Ethics Committee Protocol AEC#027/14). C57BL/6J mice (derived from Jacksons Lab, CT USA) were obtained from ARC (Western Australia, Australia) and bred at the EMSU mouse facility (Fitzroy, Victoria, Australia). GFS was performed on C57BL/6J WT and Nox4 KO male mice (10 weeks old) as previously described [3]. Briefly, mice were anesthetized using intraperitoneal injection of ketamine (100 mg/kg) and xylazine (10 mg/kg). The conjunctiva of one eye was dissected to create a small filtration space in the subconjunctival. An incision was then made with a 30-gauge needle through the sclera into the anterior chamber of the eye to create a fistula, which allows aqueous humor to exit from the anterior chamber and into the subconjunctival space. The dissected conjunctiva was then sutured over the newly created fistula. Topical antibiotic ointment Chlorsig (0.5%) was applied to the operated eye to avoid any infection after surgery. The non-operated fellow eye did not receive topical Chlorsig and served as the control. Eyes were then harvested at day-14 post-surgery to evaluate collagen deposition around the bleb. Eyes were enucleated and fixed in 4% paraformaldehyde (PFA; ProSciTech, Kirwan, Queensland, Australia) overnight, followed by agar and paraffin-embedding. Four-μm sections were then stained with Picro-Sirius red for quantification and assessment of collagen matrix in Nox4 KO and WT mice. Images were captured with a microscope (20X objective magnification, Olympus, South Australia, Australia). The area of collagen accumulation at the surgical wound was delineated and measured using ImageJ software (National Institutes of Health, Bethesda, MD, USA), by a researcher who is masked to the mouse genotype.

### 2.10. Statistical Analysis

All values are expressed as mean ± S.E.M. The mean results were analyzed using one-way analysis of variance (ANOVA) followed by post-hoc Tukey analysis. A *p* value < 0.05 was considered as statistically significant.

## 3. Results

### 3.1. TGFβ1 Cause Increase in Nox4 mRNA, H_2_O_2_ Generation, and Collagen Synthesis in HTFs

**Figure 1:**
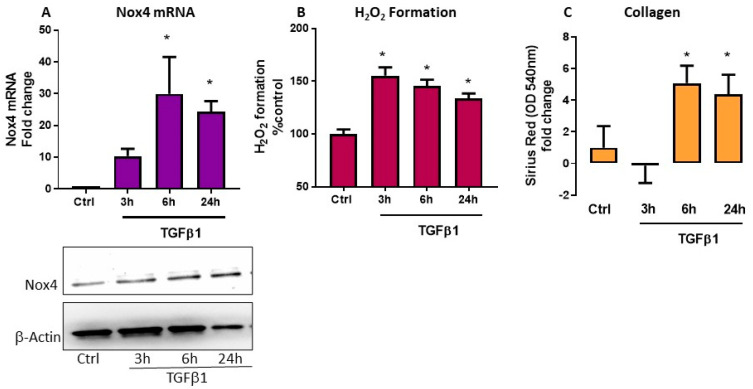
Effect of transforming growth factor (TGF)β1 in human Tenon’s fibroblasts (HTFs). Responses were determined in control fibroblasts without and with TGFβ1 (5 ng/mL) treatment for 3, 6, and 24 h. TGFβ1 caused increase in (**A**) Nox4 mRNA and protein expression and (**B**) H_2_O_2_ generation. (**A**) A representative blot and corresponding bar graph showing expression of Nox4 and internal control β-actin. (**C**) Collagen accumulation was determined by the absorbance values of Picrosirius red determined at 540 nm and TGFβ1 increases absorbances at 6 and 24 h. * *p* < 0.05 (*n* = 3–5). All data are mean ± SEM from three to five independent experiments, * *p* < 0.05 from Ctrl. without treatment.

We treated HTFs with TGFβ1 (5 ng/mL) and assayed the mRNA expression of Nox1, Nox2, Nox4 and Nox5 by rt-PCR at 3, 6, and 24 h after TGFβ1 application. Of the four Nox isoforms, TGFβ1 induced Nox4 mRNA and protein expressions at all timepoints and peak responses were seen at 6 and 24 h (Figure 1A, *p* < 0.05, *n* = 4). TGFβ1 did not alter the mRNA expression of Nox1 and Nox2 but downregulated Nox5 (data not shown). In parallel to an upregulation of Nox4 mRNA and protein, the level of H_2_O_2_ generation was elevated at all timepoints (Figure 1B, *p* < 0.05, *n* = 3–5). We assessed collagen production by evaluating the absorbance readings of the eluents from HTFs stained with picrosirius red. There was statistically significant accumulation of collagen in HTFs at both 6 and 24 h following TGFβ1 stimulation (Figure 1C, *p* < 0.05, *n* = 3–5).

### 3.2. TGFβ1-Induced Nox4 Expression Requires Smad3-Activation in HTFs

**Figure 2:**
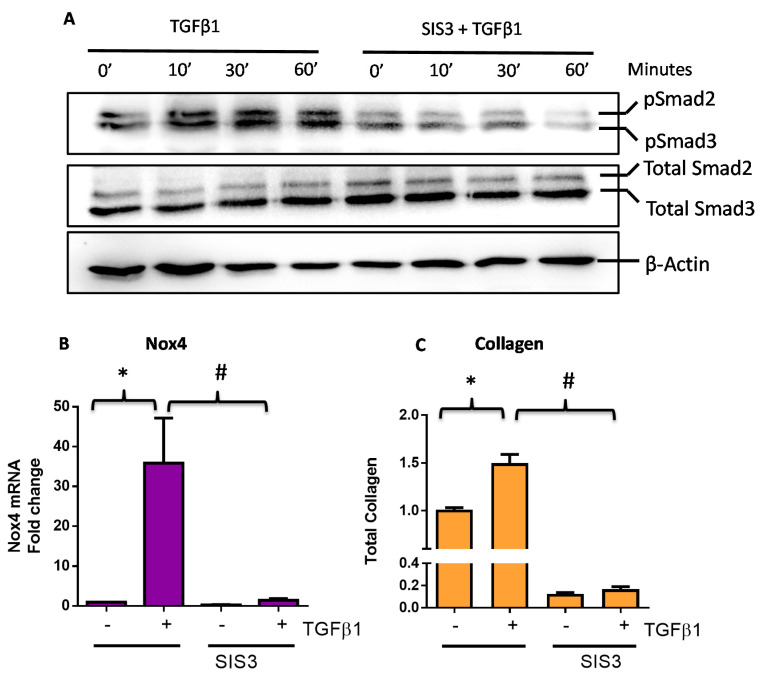
TGFβ1 responses are mediated via a Smad3-dependent pathway in human Tenon’s fibroblasts (HTFs). (**A**) A representative Western blot showing TGFβ1 (5 ng/mL) induced Smad3 phosphorylation in the absence and presence of a Smad3 inhibitor SIS3 (5 μM). (**B**) Pre-treatment of Smad3 inhibitor SIS3 suppressed TGFβ1-induced (**B**) Nox4 gene expression (6 h) and (**C**) total collagen expression (24 h). All data are mean ± SEM from three to four independent experiments, * *p* < 0.05 from Ctrl without treatment; # *p* < 0.05 from cells treated with TGFβ1.

We have previously demonstrated that the phosphorylation of Smad3 is involved in the TGFβ1-induced Nox4 gene expression and collagen production in rabbit conjunctival fibroblasts [10]. We analyzed the effect of TGFβ1 on protein expression of Smad3 (total Smad) and its phosphorylated form (*p*-Smad3) in HTFs using Western blots at 10, 30, and 60 min after treatment. As expected, TGFβ1 induced phosphorylation of Smad3 within 10 min in these cells (Figure 2A), and treatment with a known Smad3 inhibitor SIS3 was found to abolish TGFβ1-induced phosphorylation of Smad3 (Figure 2A). HTFs pre-treated with SIS3 abolished TGFβ1-induced Nox4 mRNA expression (Figure 2B) and total collagen production (Figure 2C), confirming that Smad3 is required for both responses.

### 3.3. Suppression of Nox4 and H_2_O_2_ Generation Decreased TGFβ1-Induced Responses in HTFs

**Figure 3:**
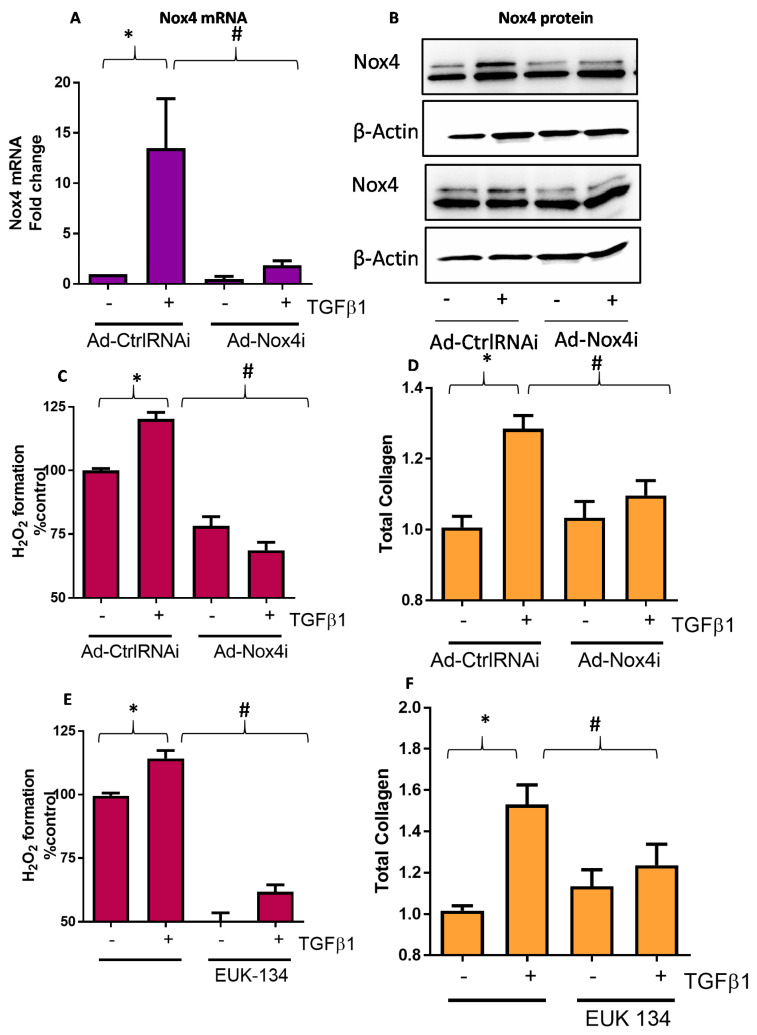
Effect of Nox4 inhibition and EUK-134 on H_2_O_2_ and collagen production in human Tenon’s fibroblasts (HTFs). Treatment of HTF with Ad-Nox4i inhibited the stimulatory effects of TGFβ1 (5 ng/mL) on expressions of both Nox4 (**A**) gene and (**B**) protein at 6 h, (**C**) generation of H_2_O_2_ at 6 h, and (**D**) total collagen at 24 h. Similarly, H_2_O_2_ scavenger EUK-134 (25 μM) inhibited TGFβ1-induced (**E**) H_2_O_2_ formation and (**F**) total collagen production. All data are mean ± SEM from three to six independent experiments, * *p* < 0.05 from control without treatment; # *p* < 0.05 from treated cells with TGFβ1.

To demonstrate the functional importance of TGFβ1-induced Nox4 expression, we used an adenoviral vector carrying siRNA targeting human Nox4 (Ad-Nox4i) to silence the expression of Nox4 in HTFs. As expected, Ad-Nox4i significantly reduced TGFβ1 induction of Nox4 mRNA (Figure 3A) and protein (Figure 3B). Importantly, we also showed that Ad-Nox4i suppressed the production of H_2_O_2_ (Figure 3C) and total collagen synthesis (Figure 3D) in the presence of TGFβ1 stimulation. Similarly, removal of H_2_O_2_ using SOD/catalase mimetic EUK-134 also suppressed TGFβ1-induced H_2_O_2_ formation (Figure 3E) and total collagen production (Figure 3F), suggesting that Nox4-derived H_2_O_2_ is required for collagen synthesis by HTFs.

### 3.4. Nox4 and H_2_O_2_ Generation Is Involved in TGFβ1-Induced Proliferation of HTFs

**Figure 4:**
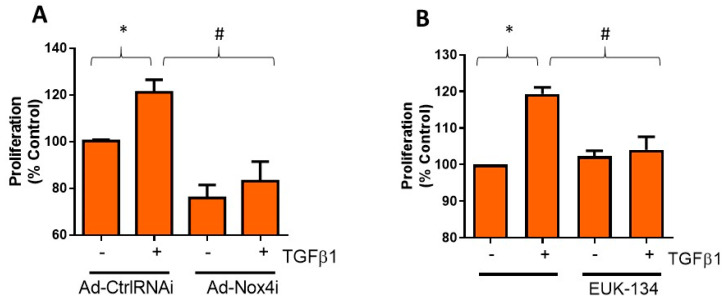
Effect of Nox4 inhibition and EUK-134 on proliferation of human Tenon’s fibroblasts (HTFs). (**A**) Treatment of HTFs with Ad-Nox4i inhibited the stimulatory effects of TGFβ1 (5 ng/mL) on cell proliferation. (**B**) Similarly, H_2_O_2_ scavenger EUK-134 (25 μM) inhibited TGFβ1-induced cell proliferation. All data are mean ± SEM from three independent experiments, * *p* < 0.05 from control without TGFβ1 treatment; # *p* < 0.05 from treated cells with TGFβ1.

Activation of Tenon’s fibroblasts is known to enhance its proliferation capacity [18] and production of fibrotic proteins, which promotes scar formation and fibrosis. We, therefore, explored Nox4 regulation on TGFβ1-induced cell proliferation of HTFs. Treatment with Ad-Nox4i markedly abolished TGFβ1 stimulated cell proliferation (Figure 4A). Ad-Nox4i was also found to inhibit cell proliferation in the absence of TGFβ1, suggesting Nox4 might be involved in cell proliferation during normal growth (Figure 4A). Likewise, scavenging H_2_O_2_ with EUK-134 suppressed proliferation of HTFs in the presence of TGFβ1 stimulation (Figure 4B).

### 3.5. Collagen Deposition at the Wound Is Reduced in Nox4-Deficient Mice with Glaucoma Filtration Surgery

**Figure 5:**
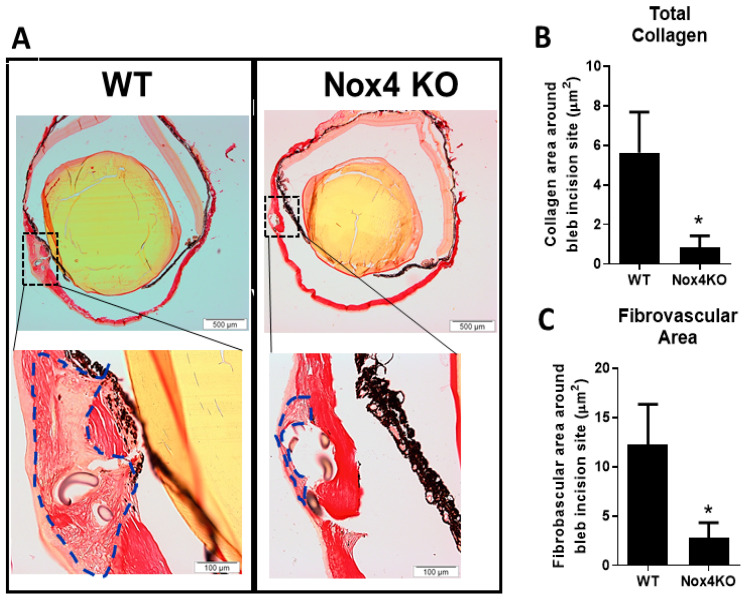
Reduced deposition of collagen at the wound in Nox4 knockout (KO) mice with glaucoma filtration surgery (GFS). (**A**) Representative images of eye cross sections from wildtype (WT) and Nox4 KO mice at 2 weeks after GFS. (**B**) A reduction in the area of both total collagen accumulation and (**C**) fibrovascularization at the wound is demonstrated in Nox4 KO mice. All data are mean ± SEM from four to five animals, * *p* < 0.05 from WT.

As expected, there are increases in both collagen accumulation (Figure 5A) and area of fibrovascularization at the wound (Figure 5B) at 2 weeks after GFS in wildtype (WT) mice. Mice deficient in Nox4 (Nox4 KO) showed a significant reduction in collagen accumulation and fibrovascular area at the wound (Figure 5A–C, *p* < 0.05, *n* = 4–5).

## 4. Discussion

Excessive post-operative scarring is a major cause of failure in GFS [19]. In this study, we demonstrate that TGFβ1-induced Nox4 expression plays an important role in collagen synthesis by using HTFs. Furthermore, we showed for the first time that accumulation of collagen at the surgical site was reduced in Nox4 KO mice at day 14 following GFS. Given the limited option for controlling post-operative scarring, targeting Nox4 could be a potential therapeutic strategy for improving long-term surgical success.

The involvement of Nox4 in TGFβ1-mediated fibrotic responses has been characterized in a variety of human fibroblasts, including cardiac [20], lung [21], and dermal [22]. To date, not much is known about Nox4 and TGFβ signaling in ocular fibroblasts in the context of eye fibrosis. Collagen synthesis by HTFs is one of the responses induced by TGFβ1 that contributes to GFS post-operative fibrosis [23,24]. We, therefore, explored the involvement of Nox4 in TGFβ1 signaling by evaluating collagen accumulation in HTFs with Sirius red assay. Sirius red assay has been validated against another collagen quantification method, like hydroxyproline assay [25], and it has previously been used to determine collagen content in rabbit Tenon [25] and fibroblast cultures [10,26]. The present study, for the first time, demonstrated that the effect of TGFβ1 on collagen accumulation in HTFs involved Nox4 expression and H_2_O_2_ generation. These findings align with our previous study that shows Nox4 is implicated in TGFβ1-mediated collagen synthesis in rabbit conjunctival fibroblasts [10] and Nox4.

Nox4 belongs to the Nox family of ROS-generating enzymes, and seven homologues, namely Nox1, Nox2, Nox3, Nox4, Nox5, and Duox1 and 2, have been identified [27]. Human fibroblasts from various tissues, such as heart [20], lungs [28], and skin [22,29], express more than one subtype of Nox. For example, pulmonary fibroblasts express both Nox1 and Nox4 [28], cardiac fibroblasts express Nox4 and Nox5 [20], and dermal fibroblasts express Nox1, Nox2, Nox4, and Nox5 [22,29]. Despite expressing different Nox isoforms, Nox4 appears to be the isoform involved in TGFβ1-mediated fibrotic responses at least in heart and lung fibroblasts [20,28]. Likewise, we found that HTFs expressed Nox4 and Nox5, and TGFβ1 stimulated Nox4 gene expression but decreased Nox5 mRNA level. This distinct effect of TGFβ1 on Nox isoforms in HTFs is similar to the findings in human cardiac fibroblasts [20].

We demonstrate that the stimulatory effect of TGFβ1 on collagen production in HTFs involved phosphorylation of Smad3. Our findings agree with the study by Xiao et al. [30], who demonstrated that inhibitor of Smad3 phosphorylation abrogated TGFβ-induced fibroblast trans differentiation and gene upregulation of collagen I in HTFs. By inhibiting Smad3 phosphorylation with SIS3, the effect of TGFβ1 on both Nox4 mRNA expression and H_2_O_2_ generation was abolished. Moreover, knocking down Nox4 gene expression with siRNA or scavenging H_2_O_2_ inhibited TGFβ1-induced collagen accumulation in HTFs. We, therefore, propose that TGFβ1 activates Nox4 expression and enhances H_2_O_2_ production through Smad3 phosphorylation and subsequently leads to collagen production. The proposed pathway is consistent with our previous findings that show TGFβ1/Smad3/Nox4 signaling mechanism in collagen synthesis in rabbit conjunctival fibroblasts [10].

To validate that Nox4 is involved in scarring responses in an in vivo model, we employed the mouse model of GFS to examine the contribution of Nox4 to collagen deposition at the surgical wound. It has been shown that the accumulation of collagen at the wound occurs in 14 days after GFS [3,31]. Further the surgery instigates a wound healing response that involves TGFβ signaling [32] and this aligns with the cell culture assays that employs TGFβ as an inducer. We demonstrated that the wound at the surgical site is packed with dense collagen fibers in WT mice with GFS. The surgical site of operated eye shows loosely organized collagen fibers and the deposition of collagen is significantly suppressed in mice lacking Nox4. This novel finding agrees with the contribution of Nox4 to TGFβ1 mediated collagen production by HTFs.

TGFβ is known to be a crucial profibrotic factor implicated in wound scarring after GFS and an antisense oligonucleotides to TGFβ has been shown to improve surgical outcome in a rabbit model of GFS [32]. Although anti-TGFβ therapy appears to be an effective way to control scarring, TGFβ has other important physiological functions, and incomplete inhibition of TGFβ likely due to insufficient dosage regimen or its receptor activation would result in severe off-target effects [9,33]. Therefore, targeting TGFβ downstream pathway may be an alternative strategy. Indeed, inhibition of TGFβ messenger protein, like Smad, has already been explored as an avenue to control conjunctival fibrosis [33]. TGFβ is found to activate Nox4 through Smad in HTF and Nox4 KO mice with GFS also show less wound scarring, our findings thus highlight the potential of Nox4 targeting in the treatment of post-surgical scarring. Further study would be required to assess if Nox4 inhibition would be efficacious in suppressing scarring responses in vivo.

## 5. Conclusions

**Figure 6:**
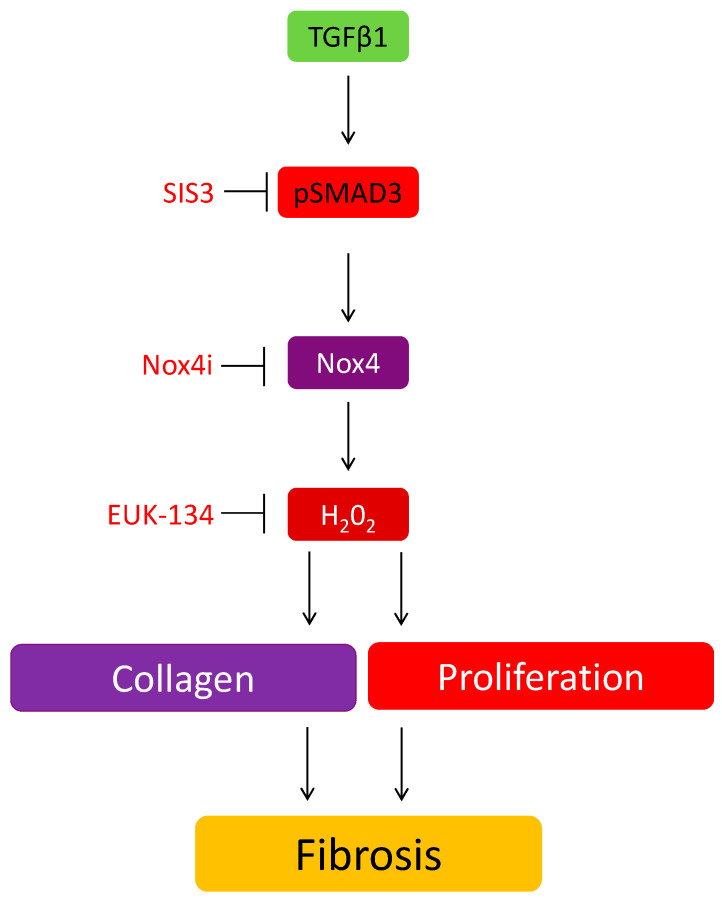
The involvement of Nox4 in the induction of TGFβ on collagen and cell proliferation in HTF.

In conclusion, we have highlighted a novel role of Nox4 in the stimulatory effect of TGFβ1 on collagen production and cell proliferation in HTFs (Figure 6). We further confirm that collagen deposition at the surgical site is significantly reduced in GFS operated mice lacking Nox4. Given the limited option for controlling post-operative scarring in GFS, targeting Nox4 would be a potential therapeutic strategy or an adjunct therapy for improving the long-term success of surgical outcome.

## Figures and Tables

**Table 1:** Human Taqman primer sequences used to amplify NADPH oxidase (Nox) isoforms for human Tenon’s fibroblasts (HTFs).

Gene Name	Gene ID	TaqMan Gene Expression Assays
Nox1	27035	Hs002455589_m1
Nox2 (CYBB)	1536	Hs00166163_m1
Nox4	50507	Hs01558199_m1
Nox5	79400	Hs00225846_m1
GAPDH	2597	4326317E

## Abbreviations

Adv-Ctrl siRNA	adenovirus expressing scrambled siRNA
Adv-Nox4i	adenovirus carrying siRNA targeting Nox
GFS	glaucoma filtration surgery
H_2_O_2_	hydrogen peroxide
HTF	human Tenon’s fibroblasts
MOI	multiplicity of infection
mRNA	messenger ribonucleic acid
NADPH oxidase 4	Nox4
PCR	polymerase chain reaction
RNA	ribonucleic acid
ROS	reactive oxygen species
SIS3	specific inhibitor of Smad3
TGFβ	transforming growth factor β
